# Phytochromes and Their Role in Diurnal Variations of ROS Metabolism and Plant Proteome

**DOI:** 10.3390/ijms232214134

**Published:** 2022-11-16

**Authors:** Markéta Luklová, Jan Novák, Romana Kopecká, Michaela Kameniarová, Vladěna Gibasová, Břetislav Brzobohatý, Martin Černý

**Affiliations:** Department of Molecular Biology and Radiobiology, Faculty of AgriSciences, Mendel University in Brno, 61300 Brno, Czech Republic

**Keywords:** diurnal, cytokinin, peroxide, phytochrome, light, signaling, glutathione metabolism

## Abstract

Plants are sessile organisms forced to adapt to environmental variations recurring in a day–night cycle. Extensive research has uncovered the transcriptional control of plants’ inner clock and has revealed at least some part of the intricate and elaborate regulatory mechanisms that govern plant diel responses and provide adaptation to the ever-changing environment. Here, we analyzed the proteome of the *Arabidopsis thaliana* mutant genotypes collected in the middle of the day and the middle of the night, including four mutants in the phytochrome (*phyA*, *phyB*, *phyC*, and *phyD*) and the circadian clock protein LHY. Our approach provided a novel insight into the diel regulations, identifying 640 significant changes in the night–day protein abundance. The comparison with previous studies confirmed that a large portion of identified proteins was a known target of diurnal regulation. However, more than 300 were novel oscillations hidden under standard growth chamber conditions or not manifested in the wild type. Our results indicated a prominent role for ROS metabolism and phytohormone cytokinin in the observed regulations, and the consecutive analyses confirmed that. The cytokinin signaling significantly increased at night, and in the mutants, the hydrogen peroxide content was lower, and the night–day variation seemed to be lost in the *phyD* genotype. Furthermore, regulations in the *lhy* and *phyB* mutants were partially similar to those found in the catalase mutant *cat2*, indicating shared ROS-mediated signaling pathways. Our data also shed light on the role of the relatively poorly characterized Phytochrome D, pointing to its connection to glutathione metabolism and the regulation of glutathione S-transferases.

## 1. Introduction

Oscillations in biological processes within a period of approximately 24 h are described as circadian rhythms. These endogenous timekeeping mechanisms are instrumental in anticipating daily environmental changes, including cycles of sunlight–darkness and temperature. The ability to cope with a wide range of daily environmental fluctuations is of the utmost importance for plants, given the limited chance to change their habitat. Examples of such rhythms can be seen in plant growth at the whole organism level and in gene expression, proteome, and metabolome regulations at the cellular level. The core clock gene network is coordinated by the transcriptional–translational feedback loops that drive rhythmic patterns throughout 24-h intervals, and a high proportion of *Arabidopsis* genes rhythmically oscillate under environmental cycles or constant conditions [1,2,3].

The rhythms in plants have been studied since the 18th century, but still hold many mysteries and promises. Reports supporting the role of the circadian clock in optimizing growth performance have been steadily accumulating [4,5], and it has been shown that the circadian clock coordinates the type and magnitude of response to key environmental factors, including drought [6], temperature [7], and biotic stress [8]. Thus, understanding how the clock works may be the key to maintaining crop yield and biomass production in agriculture under global climate change. It is also important to note that the relationship between the clock and the abiotic stimuli is not unidirectional. For example, nutrient availability can alter the circadian clock [9,10], a heat-inducible protein has been shown to repress the clock gene *PRR7* in *Arabidopsis* [11], and the expression of the clock gene was also significantly altered in *Glycine max* under drought stress [12].

Genomics studies of diurnal and circadian rhythms have been extensive, including post-transcriptional control [13,14]. However, a large-scale proteome profiling of plant rhythms has been mostly neglected. Optimistic estimates based on the available results indicate that differences in protein concentrations are only 30–40% attributable to the mRNA level [15]. The available data from published proteomics analyses confirm this, including the gel-based analyses of rice seedlings and *Arabidopsis* [16,17], the *Arabidopsis* phosphoproteome [18], the comparative analysis of selected *Arabidopsis* circadian clock mutants in two points of time [19], and the *Arabidopsis* mature plant response to a shift in photoperiod [20]. A similar setup following the light-to-dark and dark-to-light transitions revealed 288 proteins that fluctuated in their abundance [21]. Recently, the isotope labeling study showed that protein biosynthesis and protein degradation rates vary between day and night [22]. In conclusion, it seems that a significant portion of protein-based oscillations originates from constitutive mRNA expression. This is not surprising as protein biosynthesis and targeted degradation are all expensive in the ATP equivalents.

Here, the day and night protein abundances were analyzed in a loss-of-function mutant in *LHY* and four mutants in phytochromes. Light is one of the essential environmental signals for most living organisms on Earth and a crucial regulator of rhythms in plants. The oscillator is entrained or synchronized by all aspects of light, including light quality, light intensity, and the length of the photoperiod [23,24]. However, the oscillations in natural light quality are difficult to achieve with the artificial lights found in the present-day state-of-the-art growth chambers. The compromises in experimental design are inherently responsible for bias, and at least some of the light-regulated mechanisms can be lost [25]. The mutant genotypes employed in this study provided the unique opportunity to elicit regulations that would otherwise be absent in the diel cycle under standard growth chamber conditions. LHY (Late Elongated Hypocotyl) is an MYB-domain-containing transcription factor and the core circadian clock component. It is part of the morning loop in circadian regulation, with peak levels occurring around one hour after dawn. Its mutation affects diurnal rhythmicity resulting in disrupted leaf movements and a photoperiod-independent flowering [26,27,28]. *The Arabidopsis thaliana* genome encodes three types of photoreceptors that detect the red/far-red ratio: Phytochrome A (PhyA), B (PhyB, PhyD, and PhyE), and C (PhyC) [25]. Representatives of each type were used in this study. The *phyA* mutant is impaired in far-red light sensing, and the hypocotyl elongation and cotyledon expansion under continuous far-red light are inhibited [29,30]. The *phyB* mutant is defective in circadian timing, including leaf movement, CO_2_ assimilation, and light-induced gene expression, and plants flower earlier than the wild type during both long and short days [31]. PhyB functions as a thermosensor [32], and the *phyB* mutant shows a high tolerance to heat stress [33]. Seedlings with a loss-of-function mutation in *PhyC* have elongated hypocotyls and less expanded cotyledons (compared to the wild type). When grown under red light, mutant plants flower early under short-day conditions and have a lengthened circadian period [34,35]. PhyD is involved in shade avoidance and in controlling the elongation growth and flowering time. It is a close homolog of PhyB, and naturally occurring *phyD* mutants indicate that it is at least partially redundant [36,37]. Interestingly, early reports showed that all phytochromes are subjects of diurnal transcriptional control [38], but that is not supported by recent studies that have found this confirmation only for the *PhyA*-expression oscillations [14,39]. However, the diel rhythm is critical for the posttranslational control that regulates the light-induced nuclear accumulation of phytochromes [40,41,42].

Taken together, the mutants used in the present study were expected to manifest some alterations in diel regulations. The study had two main aims: (i) the identification of novel and previously unknown protein targets of the diel control and (ii) the evaluation of the role of different types of phytochrome in the regulation of proteome rhythms.

## 2. Results

### 2.1. Mutant Plants Were Slightly Paler, but Did Not Display Significant Differences as Compared to Col-0

**Figure 1:**
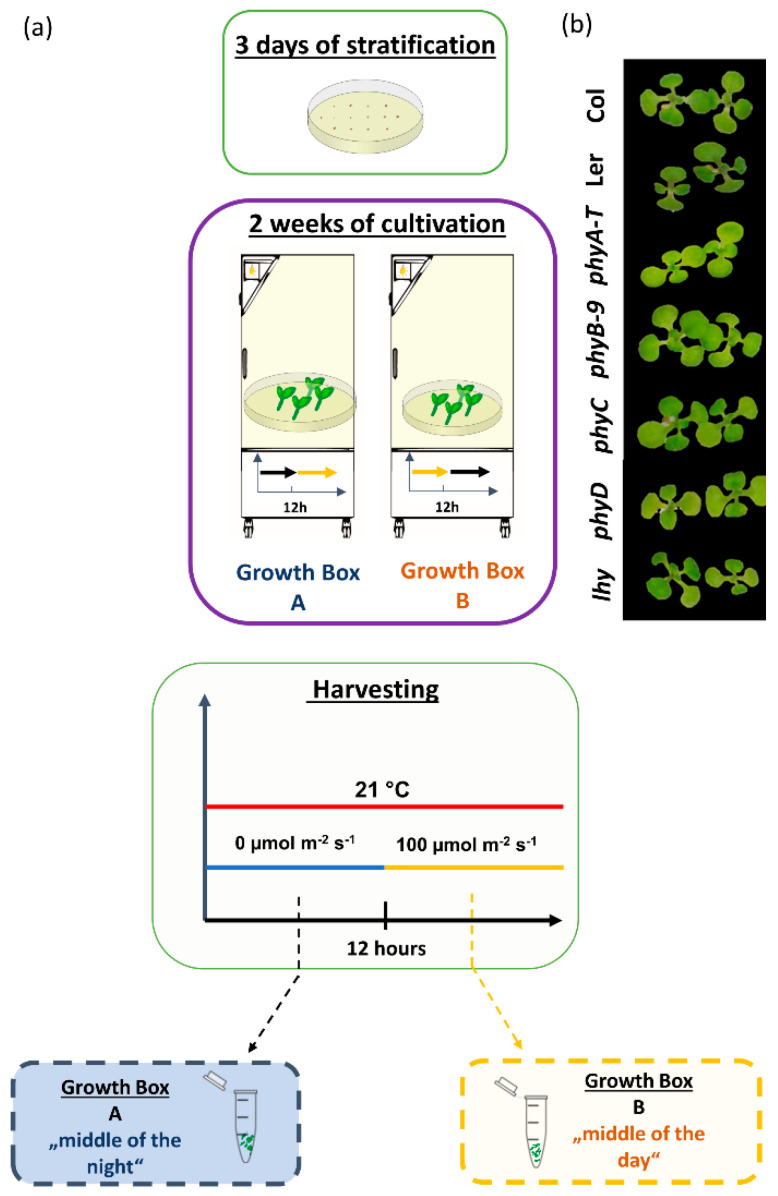
The experimental design (**a**) and representative images of two-week-old plantlets (**b**).

Mutants *phyA*, *phyB, phyC*, *phyD*, *lhy,* and the *Arabidopsis thaliana* accessions Columbia (Col-0) and Landsberg *erecta* (Ler) were cultivated as described in the Materials and Methods and outlined in Figure 1a. The night–day variations in the plant proteome were captured by analyzing two parallel sets of genotypes cultivated under the 12-h light/12-h dark photocycle with a 12-h period shift between the two sets. The plants were collected in the middle of the dark period (night) and the middle of the light period (day) (Figure 1a). The phytochrome mutants and the mutant in *LHY* were paler compared to Col-0, but otherwise showed a phenotype indistinguishable from that of wild type Col-0 (Figure 1b).

### 2.2. Plant Analysis Did Not Show Striking Differences in Total Proteome Composition

**Figure 2:**
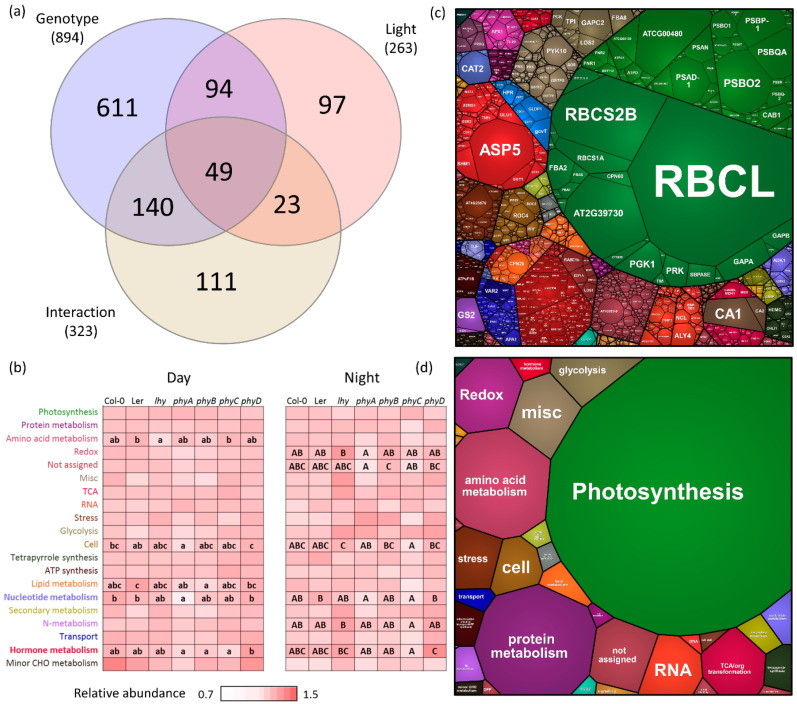
The proteome profile comparison of plantlets collected in the middle of the light and dark periods. (**a**) The results of the two-way ANOVA analysis based on 2587 quantified proteins and visualized in a Venn diagram; (**b**–**d**) Visualization of the seedling proteome in the ProteoMap; (**b**) Differences in the twenty most abundant categories visualized on a heat map and two levels of the ProteoMap (**c**,**d**). The ProteoMap visualization corresponds to the estimated content in Col-0 collected in the middle of the light period. The letters represent significant differences (*p* < 0.05, ANOVA, Tukey’s HSD). For details, see Supplementary Table S1.

**Figure 3:**
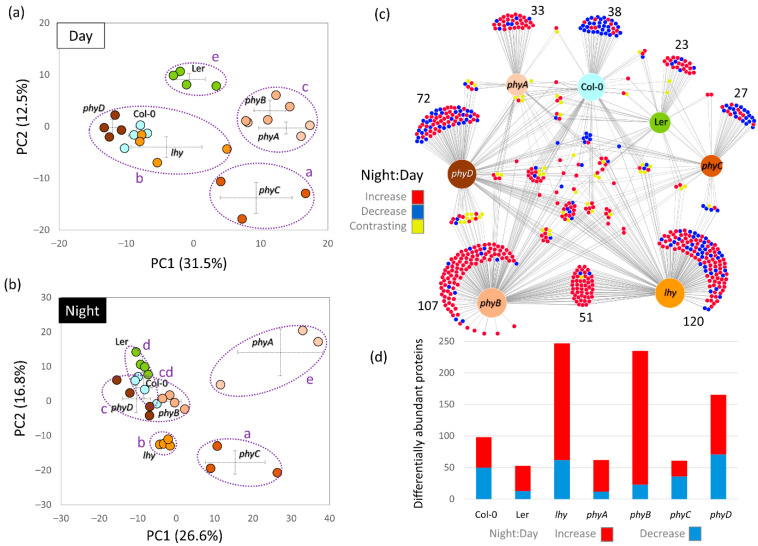
Night–day differences in proteomes of collected plantlets. (**a**,**b**) The principal component analysis (PCA) based on the profile of 315 and 650 differentially abundant proteins found in plantlets collected in the middle of the light and dark periods, respectively (ANOVA, *p* < 0.05, at least 1.5-fold change); (**c**) Comparison of the responses to light visualized with DiVenn 2.0; (**d**) Light-responsive differentially abundant proteins found in all genotypes. The results are based on at least three biological replicates. The letters in panels (**a**,**b**) indicate statistically significant differences (Kruskal–Wallis test, *p* < 0.05).

The proteome analysis of *Arabidopsis* plantlets provided the identification and quantitation of 3795 and 2587 proteins, respectively. The ANOVA analysis found more than 1000 significant differences, and most of these were related to genotype (Figure 2a). Interestingly, the observed differences were predominantly found between mutants, and the pairwise comparisons of mutants with the Col-0 proteome showed that the impact of mutations on the estimated protein content of the major protein categories was low (Figure 2b–d). Significant differences (*p* < 0.05) were found only in the nucleotide metabolism and the cell metabolism of the genotype *phyA* (day; Figure 3b). The differences between mutant proteomes were more pronounced. In addition to cell metabolism and nucleotide metabolism, the main categories showing significant differences included amino acid metabolism (day), lipid metabolism (day), hormone metabolism (day and night), redox (night), and N-metabolism (night). 

### 2.3. Night–Day Variation in Plant Proteomes Highlighted Differences in Mutants

Both the ANOVA and ProteoMaps confirmed that photoperiod had a significant impact on the observed changes in protein abundances (Figure 2a,b). The total proteome composition of mutants *phyA* and *phyC* showed the highest divergence from that of Col-0 in the middle of the light period, and *phyD* and Ler were the most similar. ProteoMap profiles of samples collected in the middle of the dark period separated *phyD* and *lhy* proteomes and showed a similarity between Col-0 and *phyB* at night. ProteoMap analyses provided a global insight into the changes in the most abundant protein categories, but were only a simplified view of the proteome. To validate the observed light-dependent differences in genotypes, all differentially abundant proteins identified (ANOVA, Tukey’s HSD, *p* < 0.05, at least a 1.5-fold change) were analyzed (Figure 3a–d). In total, 817 differentially abundant proteins were found (Table S1). The analysis showed that the absence of light stimulus had a more significant effect on the mutants (a two-fold increase in the number of differentially abundant proteins), confirming the separation of the proteome of *phyA* and *phyC* from that of Col-0, and the similarity between Col-0 and *phyB* (night) indicated by the ProteoMap analyses (Figure 3a,b).

Subsequently, the night–day protein ratios in the identified differentially abundant proteins were evaluated and 640 significant differences were found (*p* < 0.05, at least 1.5-fold change), representing almost 20% of the estimated protein content in Col-0. The comparison revealed surprisingly little overlap in the diurnal changes of protein abundance (Figure 3c,d). There were 98 significant differentially abundant proteins in Col-0, but the diurnal regulation of 38 of these seemed to be lost in the mutants. The mutants *phyA* and *phyC* that showed the most striking differences in proteome profiles compared to other genotypes (Figure 3a,b) had an attenuated diurnal regulation with only 62 and 61 differentially abundant proteins, respectively. The highest number of the night–day differences was found in the *phyB* and *lhy* mutants, indicating that these two genes are fundamental in the diurnal regulation of protein abundances. Interestingly, a high number of differentially abundant proteins was found in the day-to-night comparison in *phyD*, a mutant that did not show significant differences in the proteome profile compared to Col-0.

### 2.4. Light-Dependent Accumulation of Protein Found in Col-0 Was Predominantly Lost in All Mutant Genotypes

**Figure 4:**
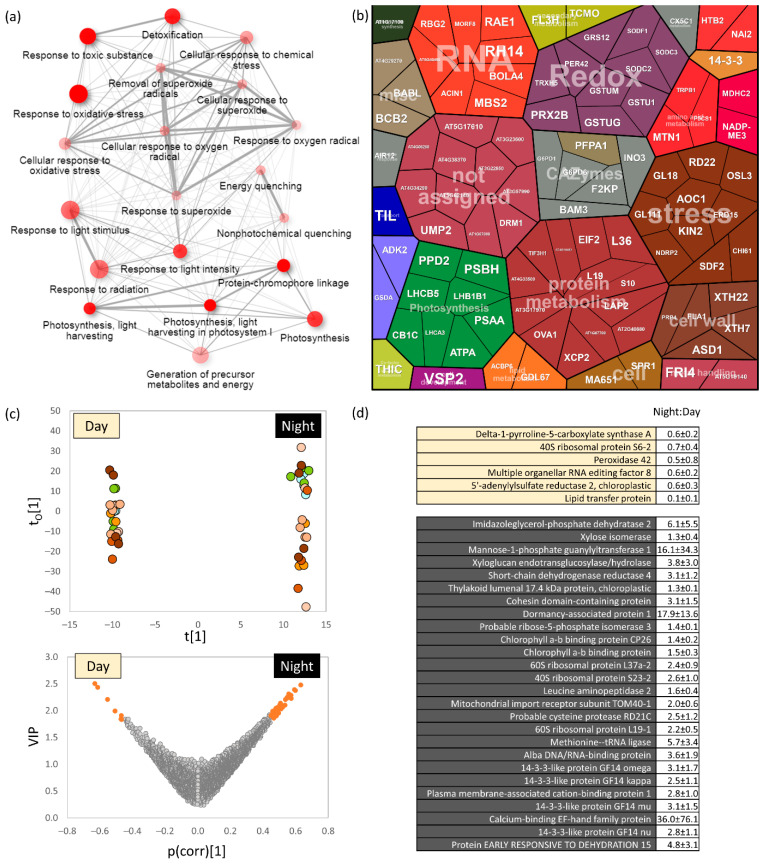
Diurnal variation in Col-0. (**a**) The most significant categories of gene ontology (GO) found in diurnally regulated proteins; (**b**) Visualization of all differentially abundant proteins (*p* < 0.05, at least a 1.5-fold change) in ProteoMap; (**c**) Identification of diurnal variations that were preserved in most mutants. The orthogonal partial least squares discriminant analysis and VIP (variable importance in projection) and (**d**) identified proteins (absolute threshold 0.5) are listed. The night–day ratio represents the mean ratio and standard deviation andcolor-coding in in the OPLS plot corresponds to Figure 3c. For details, see Supplementary Table S1.

The set of Col-0 proteins that showed significant differences in the night–day abundance ratio included the expected representatives of the photosynthetic pathway, the response to light, and ROS detoxification (Figure 4a). There were also enzymes of amino acid metabolism, energy metabolism, carbohydrate-active enzymes (CAZymes), and proteins associated with the maintenance and production of the cell wall (Figure 4b). The OPLS analysis showed that the dark-dependent protein accumulation was mostly preserved in the analyzed mutants (Figure 4c,d). However, only six Col-0 proteins that accumulated in the middle of the day showed a similar response in mutant genotypes. Nine proteins whose dark-induced accumulation was lost in the mutants included a glutaredoxin (AT4G08280; with a putative role in redox signaling) and beta-amylase BAM3 (AT4G17090; required for the starch breakdown in leaves during the night, [43]). Proteins of interest in the set of 29 proteins that were accumulated only in Col-0 in daylight included the jasmonate biosynthetic enzyme AOC1 (Allene oxide cyclase, AT3G25760), glutathione-S-transferase U16 (GSTUG, AT1G59700), thioredoxin TRXH5 (AT1G45145), and lipocalin TIL (AT5G58070); the latter is reported to play a role in thermotolerance and protection from oxidative stress [44,45].

### 2.5. Mutation in the PhyB-Modulated Accumulation Patterns of Proteins Involved in Both Primary and Secondary Metabolism

**Figure 5:**
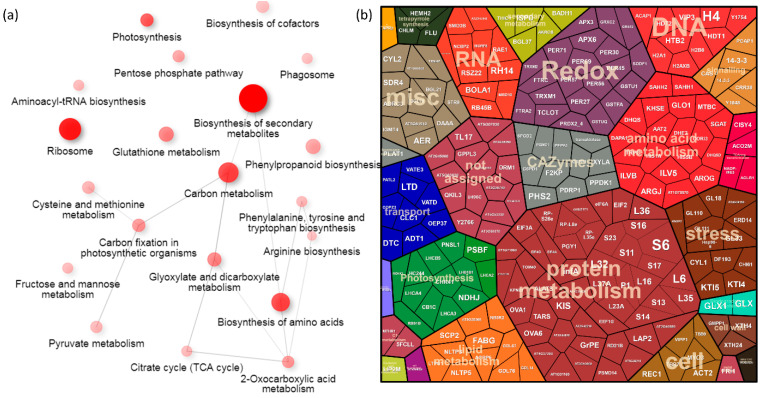
Diurnal variation in *phyB* mutant. (**a**) The most significant metabolic pathways found in diurnally regulated proteins and (**b**) visualization of all differentially abundant proteins (*p* < 0.05, at least a 1.5-fold change) in the ProteoMap. For details, see Supplementary Table S1.

The impact of a loss-of-function mutation in *PhyB* on the night–day ratio in protein abundances was the most significant of all genotypes tested in this study. Compared to its Col-0 background, only 24 of 98 regulations were not significantly affected by the mutation. Three proteins showed a contrasting response (germin GL18, AT4G14630; malic enzyme NADP-ME3, AT5G25880; and glutathione-S-transferase GSTU1, AT2G29490), and 207 regulations were not found in Col-0. Interestingly, most of these regulations were found only in the *phyB* and lhy genotypes (Figure 3c). The comparison of relative protein abundances in the Col-0 and *phyB* proteomes showed that the observed increase in the night–day ratios in *phyB* was not only a result of a dark-dependent protein accumulation. In total, only 58 *phyB* proteins showed a 1.5-fold increase in their abundance in the dark (compared to Col-0), and 88 were less abundant in the light (a 1.5-fold decrease compared to Col-0). The analysis of the metabolic pathway enrichment using KEGG annotations showed that the differentially regulated proteins in *phyB* were enriched in proteosynthesis, photosynthesis, redox metabolism, amino acid biosynthesis, and biosynthesis of secondary metabolites (Figure 5a). The ProteoMap visualization showed an overlap with the categories found in Col-0, but *phyB* had a significantly higher proportion of the categories of lipid metabolism, DNA metabolism, transport, and signaling (Figure 5b). Proteins of interest that showed *phyB*-specific regulation included glucosidase BGL18 (AT1G52400, accumulated in the night; regulates abscisic acid by releasing its active form from glucose conjugates; [46]), protein REC1 (AT1G01320, accumulated in the day; regulates the size of the chloroplast compartment; [47]), Cap Binding Protein NCBP2 (AT5G44200, accumulated in the night; mutant hypersensitive to abscisic acid; [48]), an importin subunit KPNB1 (AT5G53480, accumulated in the night; a negative regulator of abscisic acid signaling; [49]), Sterol carrier protein SCP2 (AT5G42890, accumulated in the night; has a role in seedling development, the mutant shows an altered level of amino acids; [50]), 14-3-3 protein GF14 (AT5G65430, accumulated in the night; has an impact on carbohydrate levels and metabolites of citric acid cycle; [51]), and protein HC244 (AT4G35250, accumulated in the night; required for photosystem II biogenesis; [52]).

### 2.6. Mutation in LHY Impacted Hormone Metabolism and Signaling

**Figure 6:**
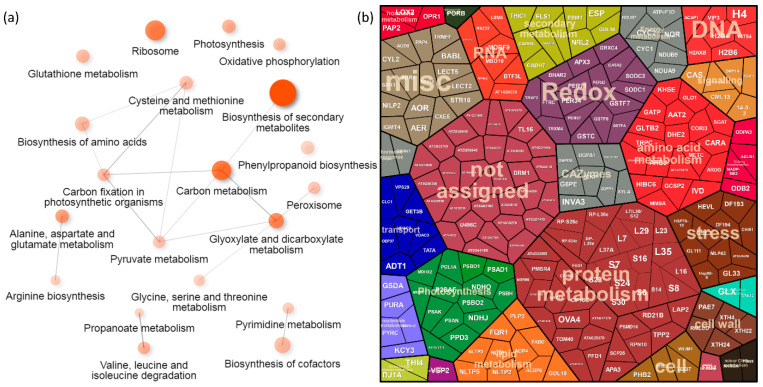
Diurnal variation in *lhy* mutant. (**a**) The most significant metabolic pathways found in diurnally regulated proteins and (**b**) visualization of all the differentially abundant proteins (*p* < 0.05, at least a 1.5-fold change) in the ProteoMap. For details, see Supplementary Table S1.

The mutation in the core gene of the circadian clock resulted in the loss of protein regulations found in Col-0, and only 13 proteins showed a similar night–day ratio to that found in the Col-0 wild type. Five proteins had an opposite regulation, including the superoxide dismutase SODC3 (AT5G18100), protein phosphatase AT4G33500, acyl-CoA-binding protein ACBP6 (AT1G31812), Basic blue protein (AT2G02850), and Photosystem II reaction center protein PSBH (ATCG00710). Most of the regulations found in lhy were not present or not significant in Col-0, and of these, 120 were found only in the lhy genotype. The ProteoMap visualization and Figure 3c highlighted the similarity between the lhy and *phyB* mutants. Accordingly, the metabolic pathway enrichment showed a similar effect on the biosynthesis of secondary metabolites, proteosynthesis, photosynthesis, glutathione metabolism, and biosynthesis of amino acids (Figure 6a). A significant enrichment absent in both Col-0 and *phyB* was found for the pyrimidine metabolism and the citric acid cycle pathway. The ProteoMap visualization showed an effect on categories of hormone metabolism, nucleotide metabolism, and energy metabolism (Figure 6b). Proteins of interest that were accumulated in the night included Prohibitin PHB3 (AT5G40770, which mediates ethylene and nitric oxide signaling [53,54]); gamma-aminobutyrate transaminase GATP (AT3G22200, which catalyzes the breakdown of the signaling molecule GABA, [55]); enzyme FLS1 (AT5G08640, which catalyzes the formation of flavonols); Phragmoplastin DRP1A (AT5G42080, which is involved in cytokinesis, vesicular trafficking, and endocytosis, [56]); and an amidase NILP2 (AT4G08790, which catalyzes the removal of a damaged deamidated glutathione, [57]). A lower night–day ratio was found for 37 lhy-specific regulations, including Cinnamyl alcohol dehydrogenase CADH5 (AT4G34230, which is involved in lignin biosynthesis, [58]); Ferredoxin-plastoquinone reductase PGL1A (AT4G22890,which is involved in cyclic electron flow); and GDSL esterase/lipase ESM1 (AT3G14210, which mediates indol-3-acetonitrile production, [59]).

### 2.7. The Alteration in Metabolism Confirms the Critical Role of ROS in Night–Day Protein Accumulation Patterns

**Figure 7:**
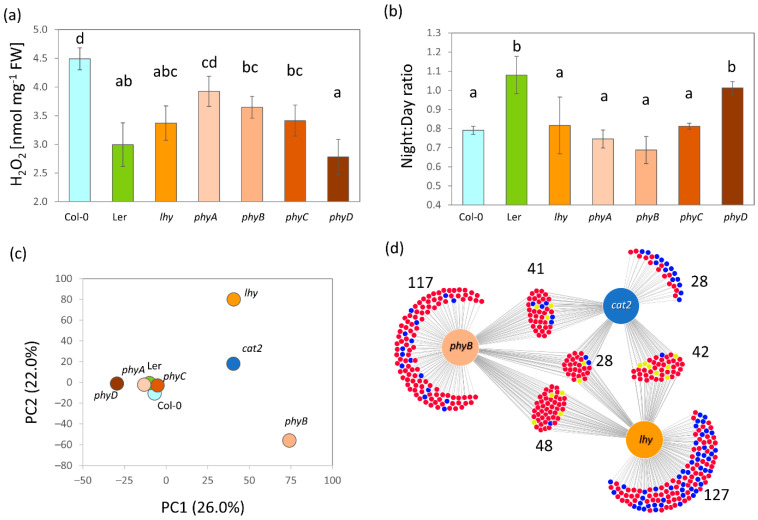
Variation in hydrogen peroxide content and effects of the catalase 2 mutation on the night–day regulation of plant proteome. (**a**) The hydrogen peroxide pool in the middle of the day and (**b**) the comparison of the night–day hydrogen peroxide content ratio in the analyzed mutants. The results are based on two complete replicates analyzed in duplicates; letters indicate statistically significant differences (Kruskal–Wallis, Dunn’s test, *p* < 0.05). (**c**) The mutation in catalase 2 showed a similarity in protein regulation to the *phyB* and *lhy* mutations. The PCA based on the identified 640 night–day regulations and (**d**) The DiVenn visualization of similarity in the observed regulations in *phyB* and *lhy*. For details, see Supplementary Table S1.

Abundances of redox metabolism enzymes were altered in all mutants analyzed (Figure 2b). For this reason, the hydrogen peroxide content was determined, and its night–day ratios were compared (Figure 7a,b). The analysis showed that Col-0 had the highest content of hydrogen peroxide, closely followed by *phyA*. The ratio between night and day hydrogen peroxide content was relatively similar for most genotypes and showed a statistically significant decrease of 20–30% (Student’s *t*-test, *p* < 0.05). The decrease was not found for the *phyD* and Ler genotypes. These two mutants had the lowest level of hydrogen peroxide (23–27% lower than Col-0), and the absence of a decrease could imply that the basal level of hydrogen peroxide had been reached. Subsequently, the catalase 2 mutant (CAT2, the night–day ratio affected by LHY, and Figure 6b) was analyzed and the effect of ROS metabolism on the observed diurnal regulations was compared. All three catalase isoforms were detected in the proteome, and this isoform was the most abundant in Col-0 (Figure 2c), representing, on average, 93 and 80% of the estimated total catalase abundance in the day and night, respectively. The mutant *cat2* showed a significant decrease in catalase 2 (9.98 ± 0.78% of Col-0), a decrease in catalase 1 (44.69 ± 24.38%), and a mild and insignificant increase in catalase 3 (111.07 ± 67.30%). The comparison of protein abundances in *cat2* and Col-0 showed that the mutant lost 85 night–day regulations, four proteins showed an opposite regulation, and only eight regulations were similar. The comparison with the whole set of differentially abundant proteins showed that 139 had significant differences (a 1.5-fold threshold, *p* < 0.05) between abundances in the middle of the day and the middle of the night.

### 2.8. Cytokinin Signaling Affects Diurnal Variation in Plant Proteome

**Figure 8:**
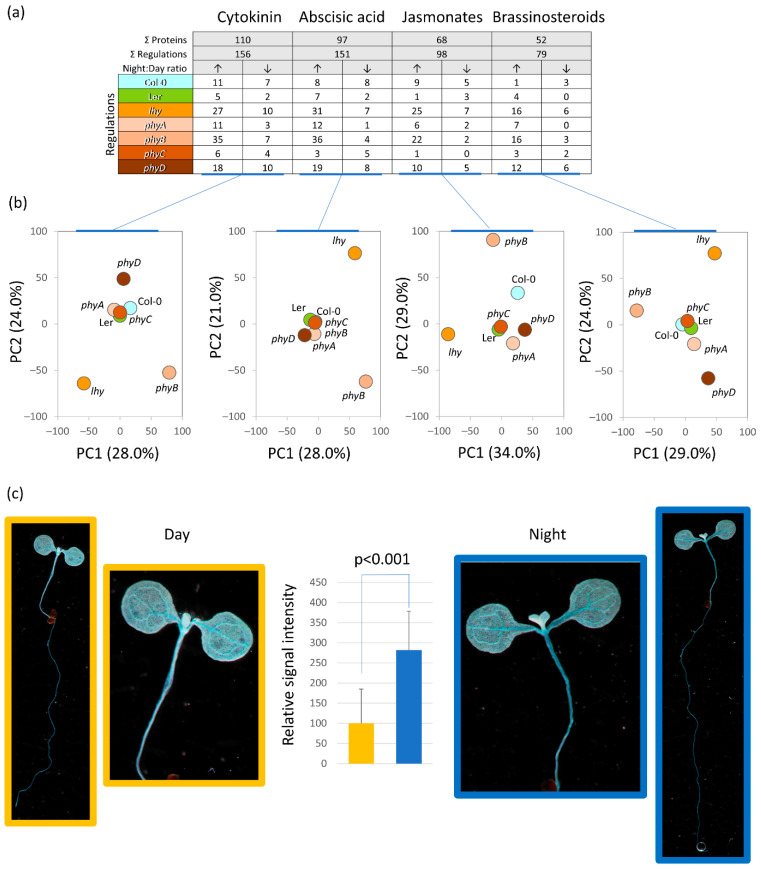
Role of phytohormones in the night–day proteome regulation. (**a**) Phytohormone response proteins found in the dataset and (**b**) corresponding PCA representations of regulations found in individual genotypes. For details, see Supplementary Table S1. (**c**) Cytokinin signaling is upregulated in the dark. Representative images of the transgenic line *ARR5::GUS* seedlings collected in the middle of the day and in the middle of the night and stained for GUS activity. The results of the quantitative analysis represent means and standard deviations of nine biological replicates, significance represents Student’s *t*-test.

The analysis of mutants indicated an alteration in the proteins related to hormonal metabolism and signaling (Figure 2b, Figure 5b and Figure 6b). A detailed comparison with previously published phytohormone-responsive proteins [60] showed that at least 261 proteins were putative targets of phytohormone signaling (Figure 8a, Table S1), including responses to cytokinin (110 proteins), abscisic acid (97 proteins), jasmonic acid (68 proteins), and brassinosteroids (52 proteins). The responses to the plant hormone cytokinin was the most numerous category, and the enzymes in the cytokinin metabolism were also part of differentially abundant proteins, including Adenine phosphoribosyltransferase 1 (APT1, At1g27450) and two adenine kinases (ADK1, At3g09820; ADK2, At5g03300). Significant differences were also observed for the component of cytokinin signaling AHP (AHP2, At3g29350; night-induced accumulation in Col-0, *phyA*, and *phyB*), but the quantitative data were based only on a single unique peptide and were not included in the final dataset. A transgenic line with a reporter for the cytokinin signaling was analyzed to provide evidence for the role of cytokinin in the observed night–day protein ratios (Figure 8a,b). The experiment showed that the cytokinin signaling output was significantly increased in the middle of the dark period, which confirmed the observed accumulation of AHP2.

## 3. Discussion

### 3.1. Identification of Novel Targets of Diurnal Regulations

**Figure 9:**
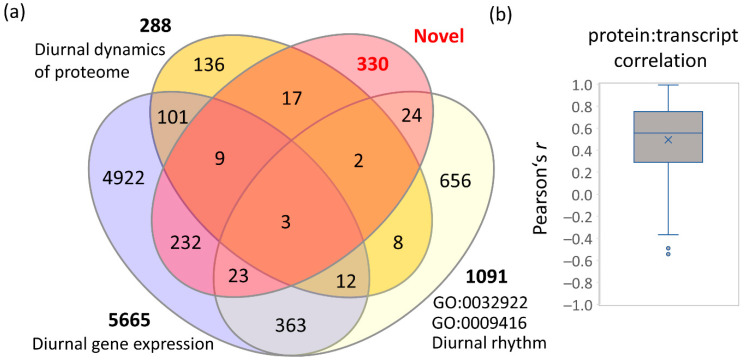
Novel targets of diurnal regulation in the *Arabidopsis* proteome. (**a**) A Venn diagram representing the overlap between the identified differentially abundant proteins and previously identified diurnally expressed genes and regulated proteins (based on https://www.arabidopsis.org/, accessed on 15 September 2022; https://www.uniprot.org/; accessed on 15 September 2022; [21,39]). For details, see Supplementary Table S1. (**b**) Pearson’s correlation between transcripts and the corresponding protein abundances. Based on data reported in the ATHENA database, accessed on 15 September 2022 [62].

The dataset obtained in the experiments described in this manuscript have provided further evidence that protein abundances are an integral factor in night–day regulations. One of the main aims of the study was the identification of as-yet-unknown components of diurnal regulatory mechanisms by employing proteome analysis and a set of mutants with a known or expected role in the diurnal oscillations. In total, 640 protein regulations were identified. The comparison with the available gene ontology annotations (https://www.arabidopsis.org/; accessed on 15 September 2022, https://www.uniprot.org/; accessed on 15 September 2022) and the two previously published large datasets of diurnally regulated transcripts [39] and proteins [21] showed that 310 of these proteins had already been associated with diurnal rhythm or a plant response to light (Figure 9a). The remaining 330 proteins have not been found in these studies, are not annotated as light-responsive or involved in diurnal rhythm, and present novel targets of natural oscillations. The comparison with diurnally regulated proteins in *Synechocystis* [39] showed that at least 34 of these proteins have a diurnally regulated ortholog (Table S1). The correlation analysis of protein abundances with transcripts found that most of these 330 proteins are not under transcriptional control (Figure 9b), and the published data has indicated that some are putative targets of post-translational control by thioredoxin [61].

### 3.2. The Observed Variations in Mutant Proteomes Could Be Associated with a Disruption in Hormonal Metabolism and Signaling

The effects of plant hormones seem to be closely regulated by the circadian clock, and hormone signaling has been proposed to represent the relay mechanism that modulates the amplitude and phase of clock output rhythms. There is emerging evidence that the clock modulates hormonal metabolism, transport, and signaling and that it determines the concentrations of phytohormones at different times of the day [63,64].

In *Arabidopsis,* the auxin biosynthetic enzyme YUCCA8 is regulated by the clock-regulated transcription factor RVE1 [65], and the clock controls the sensitivity of the plant to auxin at both the transcription level and at the stem and lateral root growth [4,66,67]. Microarray studies have also revealed a significant overlap between clock-controlled transcripts and methyl jasmonate and abscisic acid [1]. The night–day regulation of jasmonate metabolism was impacted in the *lhy* mutant (Figure 6b), and at least 68 identified proteins were previously found in response to jasmonates or oxylipins (Figure 8a). Abscisic acid accumulation oscillates with a peak in the evening, followed by the culmination of its signaling pathway components [18,68]. This concurs with the night–day regulations found in this study. In total, 97 putative abscisic acid response proteins and 151 significant regulations were found in the dataset. Of these, 116 showed significant accumulation in the night period (Figure 8a). Some regulations were shared among multiple genotypes, including an accumulation of the plasma membrane-associated cation-binding protein 1 (AT4G20260, which accumulates in response to abscisic acid, [69]). Interestingly, most of the regulations seemed to be genotype-specific, indicating a possible role of the mutated genes in the coregulation of the abscisic acid signaling output.

The cytokinin response regulators ARR6 and ARR7, cytokinin dehydrogenase, and several response factors are regulated by the clock [70], and the levels of the cytokinin pool in tobacco leaves vary diurnally, with the main peak occurring around midday [71]. On the other hand, ARR3 and ARR4 play a role in the control of the circadian period, and plants deficient in cytokinin display a highly similar expression of clock output genes to that of clock mutants. Additionally, a reduction in cytokinin status or sensitivity promotes circadian stress [72,73,74]. Here, the cytokinin response proteins were the most numerous in the identified differentially abundant proteins (Figure 8a), and a significant increase in cytokinin signaling in the middle of the night was confirmed by monitoring the output of cytokinin signaling (Figure 8c). The proportion of cytokinin-responsive proteins was particularly high in the *phyB, phyD,* and *lhy* mutants. The effect of *phyB* mutation is well-aligned with previous reports showing that the cytokinin signaling components *(ARR1*, *ARR10*, and *AHP5*) show a strong positive correlation with *PhyB* expression [75]. Cytokinin promotes *LHY* expression [76], and thus the mutation in this gene is likely to influence cytokinin-responsive proteins. Furthermore, cytokinin signaling is directly impacted by the sensor histidine kinase CKI1, which is regulated by Circadian Clock Associated 1 (CCA1, [77]). CCA1 and LHY are partially redundant and bind to the same regions of promoters [78]. It is thus possible that the loss of function mutation in *LHY* promoted *CCA1* expression and resulted in the observed regulations of the cytokinin-responsive proteins.

### 3.3. ROS Metabolism Oscillation and Its Role in the Regulation of Plant Proteome

The analysis of expression profiles showed that *PhyA*, *PhyB*, and *PhyC* share expression patterns with the hydrogen peroxide metabolism genes [79] and it is well known that the ROS homeostasis is regulated by diurnal cycles with the hydrogen peroxide production peaking at noon [80]. Our analysis showed that all mutants had a decrease in hydrogen peroxide content compared to Col-0 (Figure 7a), but the night–day oscillation seemed to be lost only in Ler and *phyD*. Interestingly, the redox metabolism enzymes were significantly enriched in all genotypes, but the individual enzymes and their isoforms were genotype-specific. Enzymes found to be significantly regulated in more than two genotypes included the superoxide dismutases (five out of eight isoforms encoded by the *Arabidopsis* genome), peroxidases, thioredoxins, and glutathione S-transferases (Table S1). The role of superoxide in circadian rhythms has been indicated in previous research [81], and only the diurnal regulation of SODC1 and SODC3 seems to be novel. The circadian regulation of the glutathione S-transferases is well-known in mammals, but our understanding of their role in plants is limited [82]. Here, 11 glutathione S-transferases were found in the list of diurnally regulated proteins. Col-0 showed a decrease in the abundances of three isoforms in the dark period (U1, U16, and U22), which is well- aligned with the previously observed diurnal changes and a decrease in these enzymes in the dark period [83]. Interestingly, none of these regulations was found in the mutant genotypes. Mutant *phyC* did not show any significant differences in the night–day abundances of these enzymes, *phyA* had an increase in isoform F12, *phyB* accumulated three isoforms (F10, U1, and U26), *lhy* genotype four (DHAR2, F10, F6, and F7), and the highest number was found in *phyD* (F2, F6, F7, U17, U22, and U26). The expression of isoform U17 is reportedly regulated by PhyA and might impact plant growth and development by interacting with auxin and abscisic acid signaling [84]. Interestingly, the content of glutathione that correlates with glutathione S-transferase activity also shows diurnal changes [85], and a recent study found that the plant hormone cytokinin has a negative impact on the glutathione pool [86]. The cytokinin signaling is upregulated in the dark (Figure 8c), and it is thus tempting to speculate that cytokinin is the master regulator behind the reported effects of glutathione S-transferases on growth and development.

### 3.4. Implications of Observed Differences and Similarities in Mutant Genotypes

*Arabidopsis thaliana* var. Landsberg *erecta* is one of the two most commonly used *Arabidopsis* accessions, and it has been used as a genetic background in many experiments [87]. Here, the experiments showed that its proteome profile was significantly different from that of the Col-0 wild type (Figure 3a,c,d). It had a much lower hydrogen peroxide content in the middle of the day. In contrast to Col-0, the hydrogen peroxide pool did not show any statistically significant differences in the dark (Figure 7a,b). Ler is not a wild type and these results contribute to the accumulating evidence that the mutation in the *ERECTA* gene has a serious impact on the molecular composition and response of the plant to the environment [88,89].

There is surprisingly little information about PhyD-specific effects on plant metabolism. Together with PhyE, it appears to fine-tune phytochrome signaling, possibly via heterodimerization with PhyB, which is considered to be the dominant class B isoform [90]. Interestingly, available data in the ATHENA database indicate that despite the higher expression level (1.6, *p* < 0.001), protein abundances of PhyB and PhyD differ only by 20% (http://athena.proteomics.wzw.tum.de, accessed on 15 September 2022 [62]). The analyses reported here show that the *phyD* mutant had a significantly altered hydrogen peroxide content (Figure 7a,b) that was unlike that of any other phytochrome mutant tested in this study. The differences to Col-0 in the night–day protein abundance ratios were not as pronounced as in *lhy* or *phyB* (Figure 3c,d), but clearly separated the mutant, indicating its role in controlling specific cellular activities. The absence of regulation in Col-0 and other phytochrome mutants included a decrease in the abundances of ribosomal proteins (AT1G18540, AT2G33450, AT4G31985, AT2G38140, and AT2G44120) and the sulfur assimilation enzyme ATP sulfurylase 2 (AT1G19920), and the accumulation of the multiple glutathione S-transferases mentioned above (Table S1).

Finally, the observed similarity between the *lhy, phyB,* and *cat2* mutants indicated a shared signaling pathway mediated by ROS metabolism (Figure 7c,d). It is known that the *lhy* mutation promotes sensitivity to oxidative stress [80] and that PhyB regulates ROS production [91]. The results reported here show that these two pathways are at least partially overlapping.

## 4. Materials and Methods

### 4.1. Plant Material

Seeds of *Arabidopsis thaliana* mutant lines *phyA-T* (N661576), *phyB-9* (N6217), *phyC* (N507004), *phyD* (N527336), and *lhy* (N531092) were obtained from the Nottingham Arabidopsis Stock Centre (Nottingham, UK). The wild-type accession Col-0 and the genotype Landsberg *erecta* (Ler-0) were obtained from Lehle Seeds (Round Rock, TX, USA). All the mutant genotypes were tested for mutation and the homozygous lines were propagated in the same bulk experiment and the seeds from that harvest were used for experiments. The *cat2* mutant was kindly provided by Dr. Pavel Kerchev.

### 4.2. Plant Growth Conditions

The seeds of the obtained mutant lines were surface-sterilized by immersion in ethanol and planted on ½ Murashige and Skoog medium solidified with 1% agar. Next, the seeds were stratified at 4 °C for 3 days. After the stratification Petri plates were transferred into growth chambers (Percival Scientific Inc., Perry, IA, USA) and cultivated at 21 °C with a 12-h photoperiod and a photon flux density of 100 µmol m^−2^ s^−1^ for 14 days. The seedlings were harvested exactly in the middle of the light or dark period, flash-frozen in liquid nitrogen and homogenized in Retsch mill (Haan, Germany). Plants for histochemical staining were cultivated as described above with the following differences: stratified seeds of *Arabidopsis thaliana* and transgenic line *ARR5*::*GUS* (obtained from The Nottingham Arabidopsis Stock Centre) were cultivated for seven days in a growth chamber (Percival Scientific) at 29 °C with a 12-h photoperiod and a photon flux density of 100 µmol m^−2^ s^−1^. On the seventh day the seedlings were harvested exactly in the middle of the light or dark period.

### 4.3. Proteome Analysis

Approximately 50 mg of homogenized tissue was extracted for omics analyses as described previously [92,93,94], and portions of the samples corresponding to 5 µg of peptide were analyzed by nanoflow reverse-phase liquid chromatography–mass spectrometry using a 15 cm C18 Zorbax column (Agilent, CA, USA), a Dionex Ultimate 3000 RSLC nano-UPLC system, and the Orbitrap Fusion Lumos Tribrid Mass Spectrometer (Thermo Fisher Scientific, Waltham, MA, USA). The measured spectra were recalibrated and searched against the Araport 11 protein database [95] and the common contaminants’ databases using Proteome Discoverer 2.5 (Thermo Fisher Scientific). The quantitative differences were determined by Minora, employing precursor ion quantification followed by normalization (total area) and calculation of the relative peptide/protein abundances. The analysis was done in at least three biological replicates (four biological replicates were collected for all genotypes; one biological replicate was lost for *phyA* and *phyC* samples).

### 4.4. Hydrogen Peroxide Determination

The determination of the hydrogen peroxide level was carried out using the PeroxiDetect™ Kit (Sigma-Aldrich, St. Louis, MO, USA) according to the manufacturer’s instructions. In brief, frozen plantlets were homogenized using a Retsch mill and 20 mg of the aliquots was extracted by 600 µL of 6% trichloroacetic acid. The hydrogen peroxide content was determined using an Infinite M1000 Pro (Tecan Inc., Research Triangle Park, NC, USA).

### 4.5. GUS Activity Staining and Quantitation

The *ARR5*::*GUS* plants were vacuum infiltrated for 10 min and then icubated in a reaction buffer containing 0.1 M phosphate buffer,1 mg mL^−1^ 5-bromo-4-chloro-3-indolyl-*β*-D-glucuronide, 0.5 mM K_3_Fe(CN)_6_, 0.5 mM K_4_Fe(CN)_6_, and 0.1% (*v*/*v*) Triton X-114; pH 7.5 was the performer for 6–8 h at 37°C. Subesequently, the leaves were bleached of the chlorophyll with a solution of ethanol (70%) andchloroform (20%) and washed with water. The plants were analyzed with a digital camera Canon, EOS 600D (Canon, Tokyo, Japan). The expression of GUS was determined by using ImageJ software [96] as described previously [97].

### 4.6. Data Analysis and Statistics

The reported statistical tests were generated and implemented as follows using the default and recommended settings unless otherwise indicated. The reliability of the protein identifications was assessed in Proteome Discoverer 2.5 (Thermo Fisher Scientific). The Student’s t-test and Pearson’s correlation were calculated using MS Excel. For the ANOVA with Tukey’s HSD and the Kruskal–Wallis tests, the Real Statistics Resource Pack software for MS Excel (Release 6.8; Copyright 2013–2020; Charles Zaiontz; www.real-statistics.com; accessed on 15 September 2022) and MetaboAnalyst 5.0 [98] were employed. The PCAs were performed in MetaboAnalyst 5.0. OPLS and the VIP was performed in SIMCA 14.1 (Sartorius, Goettingen, Germany). Significant differences refer to *p* < 0.05, unless otherwise stated. The protein functional annotations were obtained by using the UniProt database (https://www.uniprot.org; accessed on 15 September 2022) and updating the ProteoMap annotations (http://bionic-vis.biologie.uni-greifswald.de/; accessed on 15 September 2022 [99]). Similarities in regulations were visualized by DiVenn (https://divenn.tch.harvard.edu/; accessed on 15 September 2022 [100]).

## Supplementary Materials

The following supporting information can be downloaded at: https://www.mdpi.com/article/10.3390/ijms232214134/s1, Table S1: Differentially abundant proteins; Table S2: All identified proteins and peptides.
